# Promoter Variation and Transcript Divergence in Brassicaceae Lineages of *FLOWERING LOCUS T*

**DOI:** 10.1371/journal.pone.0047127

**Published:** 2012-10-11

**Authors:** Jing Wang, Clare J. Hopkins, Jinna Hou, Xiaoxiao Zou, Chongnan Wang, Yan Long, Smita Kurup, Graham J. King, Jinling Meng

**Affiliations:** 1 National Key Laboratory of Crop Genetic Improvement, Huazhong Agricultural University, Wuhan, People's Republic of China; 2 Department of Pathology, The University of Melbourne, Victoria, Australia; 3 Rothamsted Research, Harpenden, Herts, United Kingdom; 4 Southern Cross Plant Science, Southern Cross University, Lismore, New South Wales, Australia; Korea University, Korea, Republic of

## Abstract

*Brassica napus* (AACC, 2n = 38), an oil crop of world-wide importance, originated from interspecific hybridization of *B. rapa* (AA, 2n = 20) and *B. oleracea* (CC, 2n = 18), and has six *FLOWERING LOCUS T* (*FT*) paralogues. Two located on the homeologous chromosomes A2 and C2 arose from a lineage distinct from four located on A7 and C6. A set of three conserved blocks A, B and C, which were found to be essential for *FT* activation by *CONSTANS* (*CO*) in *Arabidopsis*, was identified within the *FT* upstream region in *B. napus* and its progenitor diploids. However, on chromosome C2, insertion of a DNA transposable element (TE) and a retro-element in *FT* upstream blocks A and B contributed to significant structural divergence between the A and C genome orthologues. Phylogenetic analysis of upstream block A indicated the conserved evolutionary relationships of distinct *FT* genes within Brassicaceae. We conclude that the ancient *At*-α whole genome duplication contributed to distinct ancestral lineages for this key adaptive gene, which co-exist within the same genus. *FT*-A2 was found to be transcribed in all leaf samples from different developmental stages in both *B. rapa* and *B. napus*, whereas *FT*-C2 was not transcribed in either *B. napus* or *B. oleracea*. Silencing of *FT*-C2 appeared to result from TE insertion and consequent high levels of cytosine methylation in TE sequences within upstream block A. Interestingly, *FT*-A7/C6 paralogues were specifically silenced in winter type *B. napus* but abundantly expressed in spring type cultivars under vernalization-free conditions. Motif prediction indicated the presence of two CO protein binding sites within all *Brassica* block A and additional sites for *FT* activation in block C. We propose that the ancestral whole genome duplications have contributed to more complex mechanisms of floral regulation and niche adaptation in *Brassica* compared to *Arabidopsis*.

## Introduction

Initiation of flowering is a key to the reproduction, evolution and survival of plants, and is determined by complex genetic pathways interacting with environmental and developmental signals. These are mediated through primary photoperiod, vernalization, gibberellin and autonomous pathways [1]–[3]. In *Arabidopsis*, *FLOWERING LOCUS T* (*FT*) expression causes early flowering independent of environmental and endogenous signals, whereas loss of *FT* function results in late flowering under long day (LD) conditions [4], [5]. *FT* plays a central and indispensable role for floral induction, as an integrator of different pathways [6]. *CO* binds a unique sequence element containing the TGTG (N2-3) ATG motif within the *FT* promoter to induce expression in *Arabidopsis* under long day conditions [7]–[9], whereas the FLOWERING LOCUS C (FLC) protein binds directly to the CArG box of *FT* intron 1 to repress transcription [10]. Three highly conserved blocks, A, B and C were found within an approximate 5 kb upstream promoter region, and the proximal block A and distant block C were found to be essential for *Arabidopsis FT* activation by CO binding [11]. Although the FT protein is expressed in leaves it is mobile within the plant, combining with a bZIP transcription factor FD to form a complex of FT/FD heterodimer in the shoot apical meristem. This then activates the floral meristem identity genes *APETALA 1* (*AP1*) and *FRUITFUL* (*FUL*) to promote flowering [7], [8], [12], [13].

The cultivated *Brassica* species belong to the monophyletic *Brassiceae* tribe within the Brassicaceae family [14]. Among the six *Brassica* crops in the U-triangle [15], *B. carinata* (2n = 34, BBCC), *B. juncea* (2n = 36, AABB) and *B. napus* (2n = 38, AACC) are allotetraploids, which originated from pairwise hybridization of the three diploid species, *B. nigra* (2n = 16, BB), *B. oleracea* (2n = 18, CC) and *B. rapa* (2n = 20, AA). Comparative mapping have demonstrated the presence of numerous chromosomal regions homologous to the *Arabidopsis* genome, which are effectively triplicated within the diploid *Brassica* species, consistent with a common hexaploid ancestor in the evolution of *Brassica* and the tribe *Brassiceae* [16]–[19]. In contrast, partial and whole genome comparative sequence analysis indicates that gene content is variable within paralogous regions, with interspersed gene losses and insertions compared with *Arabidopsis* [19]–[22]. Interestingly, less than 10% of coding sequences of predicted gene models from *Arabidopsis* were recently found to be retained as systemic orthologues in each of the triplicated A subgenomes (*Brassica rapa* Genome Sequencing Project Consortium [23]).

Transposable elements (TEs) are ubiquitous amongst eukaryotic genomes. Of the most prevalent classes, retroelements and DNA elements [24] are believed to be major drivers of genome and gene evolution [25]–[27]. In plants, most genome size variability is associated with differences in repetitive DNA content, primarily ascribed to differential amplification of TEs [28]. The CACTA element of the En/Spm transposon system was first isolated and characterized at the molecular level in maize [29], and is estimated to be the most abundant DNA transposon family in *B. oleracea* [30]. *B. oleracea* transposon 1 (*Bot1*), a C genome-specific CACTA element, is believed to play a major role in the recent divergence of the *B. rapa* and *B. oleracea* genomes [31]. In *Arabidopsis*, the major effect of TE DNA methylation is to silence transposition. However, methylated sequences may also affect the transcription of the flanking genes, typically reducing their expression [32]–[34].

We previously identified six *BnFT* paralogues in *B. napus* [35], and showed that four of these were located within inverted duplicated blocks of homoeologous sections of chromosomes A7 and C6 (*BnC6.FT.a* and *BnC6.FT.b*; *BnA7.FT.a* and *BnA7.FT.b*). The remaining two paralogues were located on homoeologous chromosomes A2 and C2 (*BnA2.FT* and *BnC2.FT*) with deletion or variation of a putative CArG box for the FLC protein binding site that had been identified in Arabidopsis within intron 1 [10]. The phylogenetic tree based on the coding sequence clearly demonstrates that *BnFT* paralogues share a common ancestor with *Arabidopsis FT*, but had undergone triplication events consistent with the hypothesis of a common hexaploid ancestor for the *Brassica* species.

Here, we report the isolation and comparison of the *FT* promoter sequences from A2 and C2 that have a common lineage, and explore their evolutionary history both within the genus and the Brassicaceae family. *FT* transcripts from the distinct A2/C2 and A7/C6 lineages were then analyzed from a sample of eleven *Brassica* diploid and tetraploid cultivars at different developmental stages and environments, revealing divergence of expression patterns. Finally, the potential regulatory mechanisms, including DNA methylation, are further explored and discussed.

## Results

### Characterization of homoeologous *FT* genes on *Brassica* A2/C2 chromosomes

**Figure 1:**
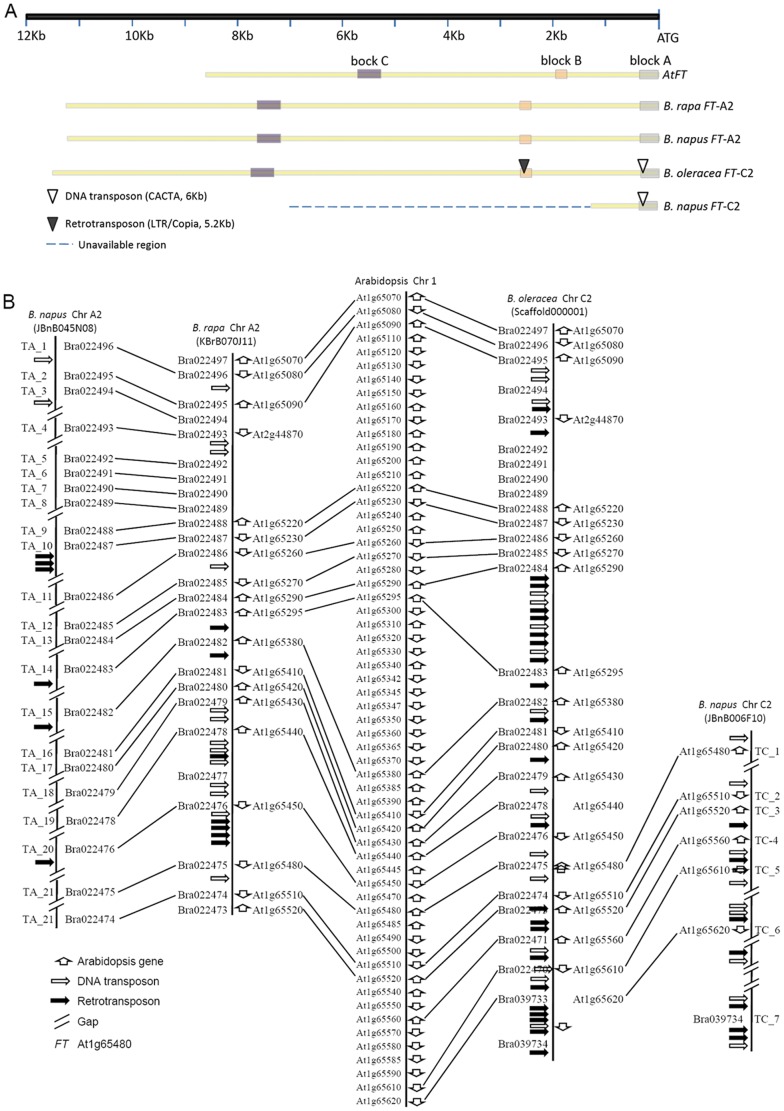
The structure of *FT* promoter and alignment of conserved genes. (A) Three conserved blocks were contained in promoter of *FT*-A2/C2 in comparison with *FT* in *Arabidopsis*. (B) The comparison map of *FT*-A2/C2 flanking fragments from *B. rapa*, *B. oleracea*, *B. napus* and *Arabidopsis*.

Our previous results indicated that *Brassica FT* orthologues on chromosome A2 and C2 arose from a common lineage [35]. We isolated the complete promoter region of *FT*-A2 and partial promoter of *FT*-C2 from two BACs of *B. napus* Tapidor DH, JBnB045N08 and JBnB006F10. Based on the established upstream blocks A, B and C required for *FT* activation in *Arabidopsis* [11], we aligned the corresponding upstream regions of *FT*-A2 and *FT*-C2 from *B. napus*, *FT*-A2 from *B. rapa* BAC KBrB070J11 and *FT*-C2 from *B. oleracea* Scaffold000001 (unpublished data, available upon request from liusy@oilcrops.cn). This demonstrated the conservation of these blocks in the *FT* upstream regions of the *Brassica* homoeologous chromosomes A2 and C2 (Figure 1A). Interestingly, a CACTA element of approximately 6 kb was found inserted within *FT*-C2 block A, and a 5.2 kb retrotransposon within block B of the same upstream region (Figure 1A). The sequences of four promoters are available in NCBI Genbank (accessions JX193765 (*FT*-A2), JX193766 (*FT*-C2), JX193767 (*FT*-C6a) and JX193768 (*FT*-C6b)).

We then analyzed micro-synteny in the genome segments containing *FT*-A2 and *FT*-C2 from *B. rapa*, *B. oleracea* and *B. napus*, as well as the orthologous region from *Arabidopsis*. This indicated that TEs were more abundant in the neighboring region of *FT*-C2 than the homologous region of *FT*-A2. Unfortunately, we had limited access to data from the corresponding regions of *FT*-C2 in *B. napus* compared with *B. oleracea* (Figure 1B). However, we did identify potential hot spots for the insertion of TEs within the intergenic region between orthologues of Atlg65290 and Atlg65295 on *Brassica* chromosome C2 (Figure 1B).

We designed specific primer sets to distinguish the upstream regions containing the transposon and retrotransposon within block A and B from the A and C genome. These were used for PCR amplification of samples from a range of accessions, including *B. rapa* (17), *B.oleracea* (17), *B. napus* (51), *B. nigra* (1), *B. juncea* (8), *B. carinata* (6) and 9 species representing a range of taxa from the Brassicaceae. Based on the evidence from these marker assays, TEs were only detected within the *Brassica* C genome, and no insertion was found in the homologous regions of *Brassica* A and B genomes, nor from other Brassicaceae (Table 1). We concluded that these transposon insertions most likely occurred after the divergence of the *Brassica* A and C genomes.

**Table 1:** Detection of TE insertion in block A and block B of Brassicaceae species.

Gene location	Number of accession with TE insertion in corresponding *FT* paralogous						
	*B. rapa*(AA)(17 accessions)	*B. oleracea*(CC)(15 accessions)	*B. napus* (AACC)(51 accessions)	*B. nigra* (BB)(1 accession)	*B.juncea* (AABB)(8 accessions)	*B.carinata* (BBCC)(6 accessions)	Wild species(9 accessions)
Block A_A2	0	/	0	0	0	/	0
Block A_C2	/	15	51	0	/	6	0
Block B_A2	0	/	0	0	0	/	6
Block B_C2	/	15	51	0	/	6	2

### Evolutionary analysis of *FT* upstream block A in Brassicaceae

**Figure 2:**
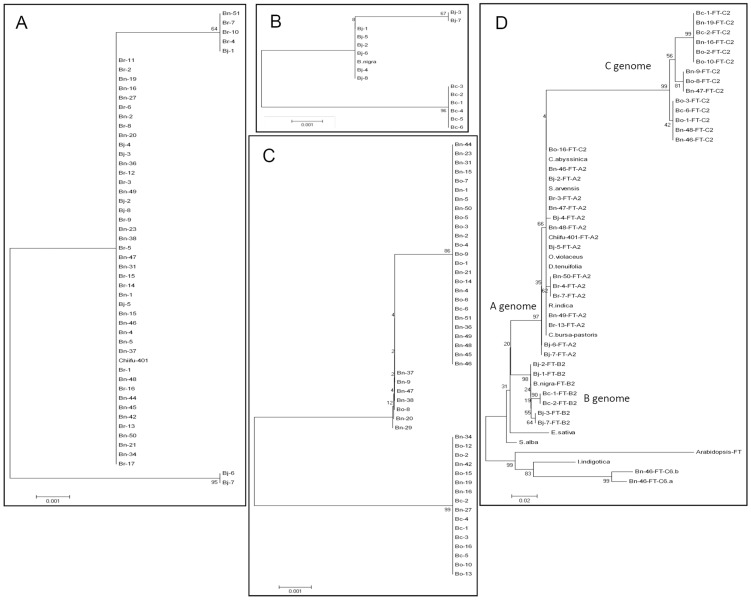
The phylogenetic relationship of *FT* block A. (A) Phylogenetic tree of block A of *FT*-A2, Br represented *B. rapa*, Bn represented *B. napus* and Bj represented *B. juncea*. (B) Phylogenetic tree of block A of *FT*-B2, which shared the same lineage with A2 origin. Bc represented *B. carinata*. (C) Phylogenetic tree of block A of *FT*-C2. Bo represented *B. oleracea*. (D) Phylogenetic tree of block A comes from a different lineage in *Brassicaceae*.

The sequences of *FT* upstream block A from a sample of diploid and tetraploid *Brassica* cultivars were determined and aligned, based on use of specific primer pairs for *FT*-A2 and *FT*-C2. The derived phylogenetic tree indicates that most *B. napus* and *B. juncea* accessions share high sequence similarity for block A with *B. rapa*, apart from two *B. juncea* accessions, Bj-6 and Bj-7 (Figure 2A). Accessions of *B. juncea* and *B. carinata* containing sequences of B genome origin were clearly allocated to two distinct groups, with only one variety of *B. nigra* grouped with *B. juncea* (Figure 2B). Three distinct groups were identified in the C genome phylogenetic tree, one including only *B. oleracea* and *B. napus*, and the other two including *B. oleracea*, *B. napus* and *B. carinata* (Figure 2C). This is consistent with the *FT*-A2/C2 lineage being conserved during the allopolyploidisaton of *Brassica* species.

In order to explore the wider evolutionary relationships of *FT* genes within the Brassicaceae, we isolated the sequences of upstream block A from 9 representative genera, using the specific primer pairs for the *FT*-A2 orthologues. The block A sequences of the *FT*-A2/C2 lineage from the diploid and tetraploid *Brassica* species and representative Brassicaceae species were aligned with those of *Arabidopsis FT*, *BnC6.FT.a* and *BnC6.FT.b*. There is a clear differentiation of block A within the inverted duplication blocks of the *FT*-A7/C6 and *FT*-A2/C2 lineages. The *Brassica FT*-A7/C6 genes had high identity with that of *Isatis indigotica* and *Arabidopsis* (Figure 2D), whilst the *Brassica FT*-A2 paralogue had higher identity with that of *Orychophragmus violaceus*, *Rorippa indica*, *Capsella bursa-pastoris*, *Diplotaxis tenuifolia*, *Crambe abyssinica* and *Sinapi arvensis*, than the corresponding *Brassica* B lineage (Figure 2D). These results strongly suggest that the distinct lineages observed within the *Brassica* genomes arose prior to the ancestral whole genome triplication events.

### Differential expression and DNA methylation analysis of *FT* genes in *Brassica* diploids and tetraploids

**Figure 3:**
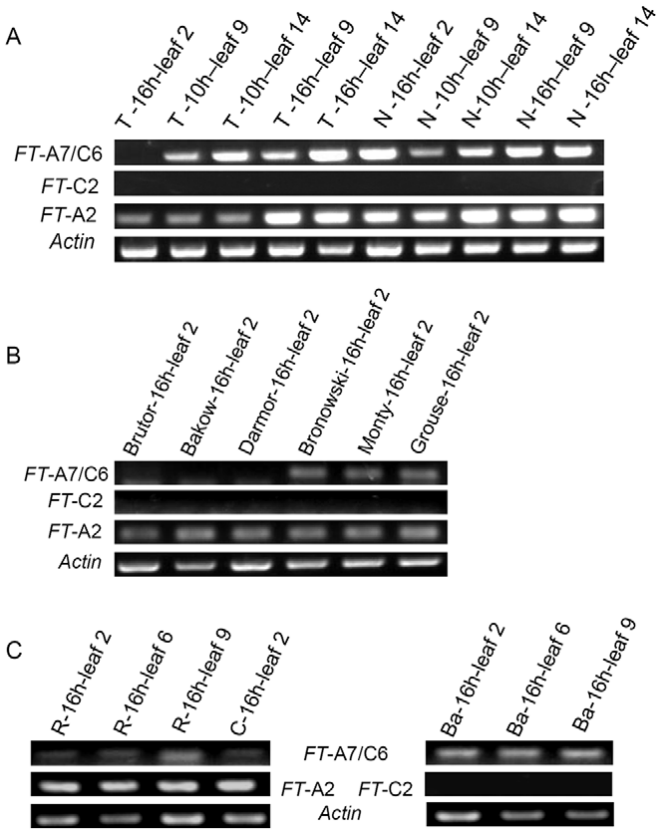
Expression Patterns of *FT* genes in leaves of *B. napus*, *B. rapa* and *B. oleracea*. (A) RT-PCR analysis of *FT* genes and *Actin* in leaves of *B. napus* Tapidor DH and Ningyou7DH under LDs and SDs. T represents TapidorDH and N represents Ningyou7DH. (B) RT-PCR analysis of *FT* genes in leaf 2 from three winter type and three spring type cultivars of *B. napus* under vernalization free and long day conditions. (C) RT-PCR analysis of *FT* genes in leaves of the lines from *B. rapa* and *B. oleracea* under vernalization free and long day conditions. R represents R-o-18, C represents Chiifu-401, and Ba represents *B. oleracea var. alboglabra*. R-o-18 and Chiifu-401 are cultivars of *B. rapa*, and *B. oleracea var. alboglabra* is a cultivar of *B. oleracea*. Note: 10 h and 16 h represent the SD and LD condition, leaf 2–14 represent the leaves collected at different development stage. Leaf 2 was collected before vernalization and leaf 6 was collected immediately after vernalization.

Since we had observed strong evidence for lineage-specific variation in upstream sequences within the paralogous *FT* genes, we then explored potential consequences on the pattern of gene expression. We designed specific primer sets to detect transcription from each of the *FT*-A2/*FT*-C2 and the *FT*-A7/C6 paralogues, using semi-quantitative RT-PCR. Within TapidorDH (*B. napus*, winter type DH line) and Ningyou7DH (*B. napus*, semi-winter type DH line) *FT*-A2 was transcribed in all leaf samples from different developmental stages and different photoperiod treatments, whereas *FT*-C2 was not transcribed (Figure 3A). Moreover, *FT*-A2 was found to be transcribed in leaf 2 from three other winter type cultivars, and three spring type cultivars under vernalization-free conditions, whilst *FT*-C2 was not (Figure 3B). Interestingly, under the same conditions *FT*-A7/C6 was not transcribed in leaf 2 of TapidorDH and three other winter type cultivars, but was transcribed in Ningyou7DH and three other spring type cultivars (Figure 3A–B).

We then checked *FT* transcription in leaves from *B. rapa* and *B. oleracea* at three different developmental stages without vernalization. *FT*-A2 was transcribed in all samples from two crop forms of *B. rapa*, yellow sarson (R-o-18) and Chinese cabbage (Chiifu-401), whereas *FT*-C2 was not transcribed in *B. oleracea var. alboglabra*, where TEs were inserted in block A and B (Figure 3C). The *FT*-A7/C6 was transcribed in leaves from all diploid accessions, although the transcript level from the C genome appeared to be higher than that from the A genome (Figure 3C).

**Figure 4:**
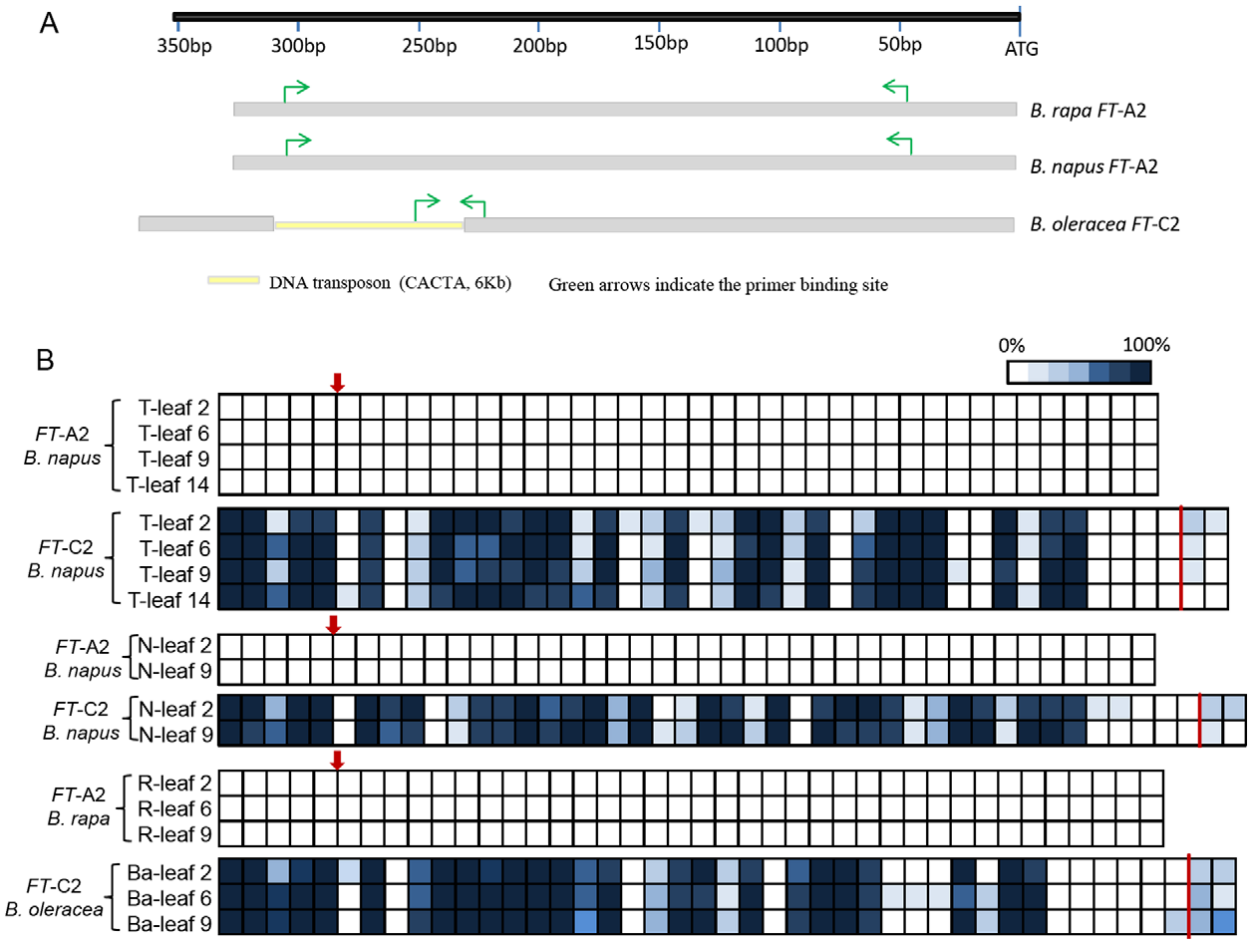
DNA methylation profiles of block A. (A) Scheme representing the location o TE insertion within blocks A of *FT* promoters on chromosome A2 and C2, together with primers used. (B) The methylation state of block A from C2 was analyzed using the DNA collected from leaves of *B. napus* and *B. oleracea* by bisulphite sequencing of ten random clones. T represents TapidorDH, N represents Ningyou7DH and Ba represents *B. oleracea var. alboglabra*. One box represents one cytosine (C) and 0–100% represents the percentage of methylated cytosine in (5^m^C) detected in the ten random clones sequenced. Red arrows: TE insertion site in the C genome; Vertical line: boundary of TE.

We were interested to determine whether the TE insertion in *FT*-C2 had a significant effect on the neighbouring DNA methylation status. Cytosine methylation analysis of bisulphite treated DNA from leaves of diploid and tetraploid accessions focused on target regions within the *FT* upstream blocks A from C2 and A2 lineages (Figure 4A). We found high levels of cytosine methylation within the TE sequence of ten randomly selected clones, and low levels in flanking sequence for *FT*-C2 block A from *B. napus* TapidorDH and Ningyou7DH, as well as from *B. oleracea var. alboglabra* (Figure 4B). However, the corresponding *FT*-A2 upstream block A that lacked the TE insertions was free of cytosine methylation for all developmental stages tested in *B. napus* TapidorDH, Ningyou7DH and *B. rapa* R-o-18 (Figure 4B).

### Regulatory motif analysis of *FT* genes in *Brassica*

**Figure 5:**
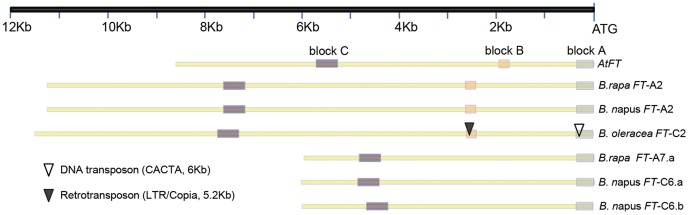
Diagram of conserved blocks within promoter of *FT*-A2/C2 and *FT*-A7/C6. Block A and block C were detected in all *FT* promoters of *Brassica* whereas block B was not detected in *B. rapa FT*-A7.a from BAC (KBrB092C03) of Chiifu-401, *B. napus FT*-C6.a and *FT*-C6.b from TapidorDH BAC JBnB104L19 and JBnB054L06, respectively.

**Figure 6:**
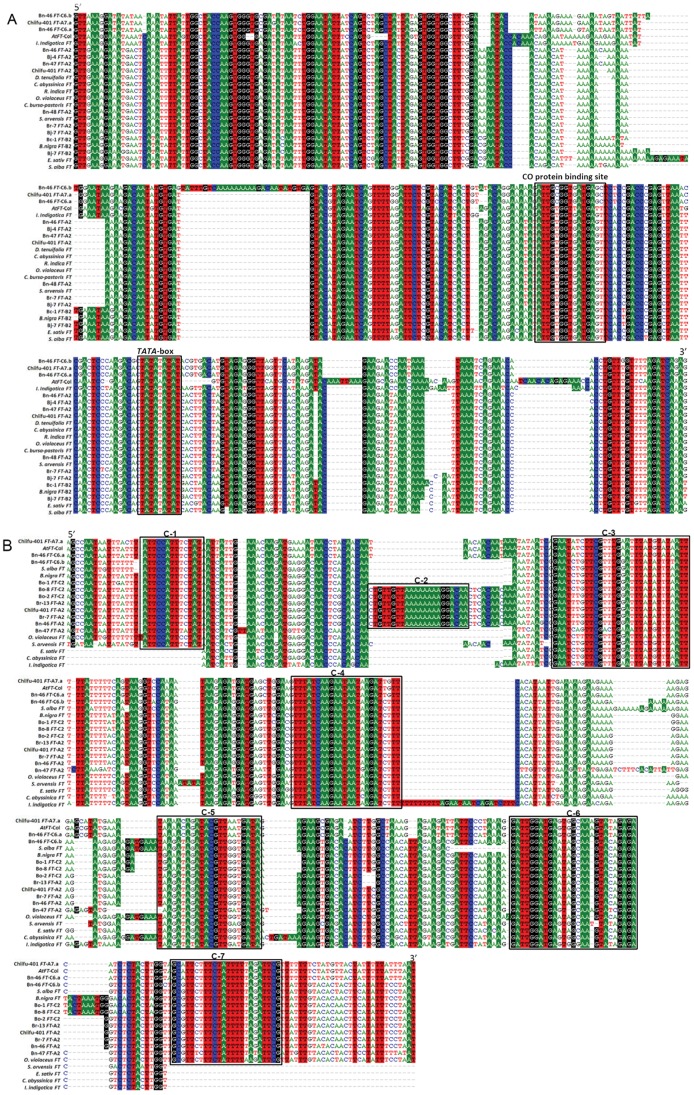
Potential motifs analysis in conserved blocks of *FT* promoter in Brassicaceae. (A) Sequence alignment of block A from different species in Brassicaceae. The putative CO protein binding site and *TATA*-box were indicated. (B) Sequence alignment of block C in Brassicaceae. Potential conserved blocks were indicated with open black box.

Comparison of promoter regions from *FT*-A2/C2 and *FT*-A7/C6 lineages indicated that the latter were shorter, and lacked the upstream block B (Figure 5). This strongly suggests that blocks A and C are more essential for the activation of *Brassica FT* genes. Block A appears to be highly conserved across different Brassicaceae genera (Figure 6A). The ability of *CO* to promote *FT* expression is well established and involves binding a consensus TGTG (N2-3) ATG motif within the *FT* promoter [4], [9]. We are now able to distinguish three distinct CO protein binding site variants within block A: type I = “ATTGTGGTTATGATT” in *Arabidopsis*, type II = “ATTGTGGTGATGAGT” in the *Brassica FT*-A2/C2 lineage, and type III = “ATTGCGGTGATGAGC” within the inverted duplication blocks of *Brassica FT*-A7/C6 lineage (Figure 6A). It is possible that these sequence variants for the binding site correspond to the multiple CO paralogues in *B. napus* [36], [37]. In contrast to the CO protein binding site, the putative TATA-box is more conserved within Brassciaceae, with only one base variation found in *Arabidopsis* (Figure 6A).

Block C has been proposed as being another essential region for *FT* activation in *Arabidopsis* and *Brassica*. We therefore isolated the upstream block C of *FT*-A2/C2 homoeologous lineage from different species within the Brassicaceae and compared them with the corresponding region from *Arabidopsis FT*, *B. napus FT*-C6.a and *FT*-C6.b and *B. rapa FT*-A7.a. The resulting alignment demonstrated that block C is highly conserved across the *Brassicaceae*, although consistently located between 4.5 and 7.5 kb upstream of the start codon. We were able to designate a series of 10–30 bp regions, highly conserved within the Brassicaceae, as potential functional motifs within block C. Of seven sites (designated C-1to C-7) identified, six were universal within Brassicaceae, and another (C-2) specific to the *Brassica FT*-A2/C2 lineage (Figure 6B). This comparative taxonomic analysis provides strong circumstantial evidence for their role as potential motifs for *FT* activation.

## Discussion

### Structure and diversification of the *Brassica FT*-A2/C2 promoter

We previously established that all extant *Brassica FT* genes share a common ancestry with *Arabidopsis FT* [35]. Here, we isolated and aligned promoter sequences from the diploid and amphidiploid representatives of the *FT*-A2/C2 lineage and demonstrated conservation of upstream blocks A, B and C previously identified in *Arabidopsis* [11] as being of key functional significance for activation of *FT* transcription, and hence flowering. However, since the divergence of the *B. rapa* A and *B. oleracea* C genomes around 4 MYA [20], [38], two class TEs have inserted specifically within *FT*-C2 upstream blocks A and B. Our micro-synteny analysis indicated that TE insertion appears generally to be more abundant in the vicinity of *B. oleracea FT*-C2 of the homoeologous regions of *B. rapa FT*-A2. This may have resulted from genome-wide TE insertion events (Figure 1B) which are more abundant in the C genome, as revealed by homoeologous BAC sequencing [38], [39]. The presence of a CACTA element within *FT*-C2 block A appears to have contributed to non-functionalization of this key floral regulator. CACTA element insertion leading to gene function diversification is common in plants [40]–[43]. For example, in soybean, a 20.5 kb insertion in the *flavonoid 3′-hydroxylase* (*F3′H*) gene led to a new allele conferring a novel phenotype of stable gray trichome color. The 20.5 kb insertion has the molecular structure of a putative autonomous transposon of the CACTA family [42].

### Conservation of *FT* upstream block A within the Brassicaceae

*FT* genes are located in genomic regions corresponding to *Arabidopsis* chromosome 1 block E [35]. In *Brassica* species, extensive comparative genomic studies have shown that copies of block E are found on chromosomes A2, B2, C2, A7, B7 and C6 [19], [44]–46. Since block E is inversely duplicated on A7 and C6 [35], [47] and there is extensive homoeology among the two sets of linkage groups A2/B2/C2 and A7/B7/C6 [45], we were confident that the large block E on B7 is also inversely duplicated. The presence of two *FT* lineages could be inferred within the B genome, with *FT*-A2/C2/B2 representing homeologues from one lineage and *FT*-A7/B7/C6 from the other.

The sequences of *FT*-A2/C2 upstream block A were grouped according to genomic content of *Brassica* diploid and tetraploid species within the U triangle (Figure 2A–C), which suggests that the *FT*-A2/C2 lineage was conserved during the process of the amphidiploid formation and domestication. A wider phylogenetic analysis of *FT*-A2/C2 and *FT*-A7/C6 upstream block A, that included a representative range of Brassicaceae species, demonstrated the clear distinction between the different groups (Figure 2D), further confirming the independent *FT* lineages. It is particularly surprising that block A of *I. indigotica* clearly grouped with the *FT*-A7/C6 lineage even though the sequence was isolated with A2 specific primers and the *FT*-A2/C2 lineage cluster was interspersed with a combination of *Brassica* species and other genera. A recent taxonomic analysis of the Brassicaceae has indicated that *I. indigotica* belongs to the *Isatideae* tribe, a neighbor of the *Brassiceae* which includes *O. violaceus*, *S. alba*, *S. arvensis*, *E. sativ*, *D. tenuifolia* and *C. abyssinica*. Meanwhile, *C. bursa-pastoris* and *Arabidopsis* belong to the *Camelineae* and *R. indica* belong to *Cardamineae* which are further removed from the *Brassiceae* [14], [48]. Although there is no ready explanation for these conflicting interpretations, it is recognized that genome evolution of the *Brassiceae* and related species is greatly complicated by the process of genome duplication, hybridization, and polyploidy. This makes it difficult to give a clearly evolutionary route for every genus and species [18], [49]. Nevertheless, phylogenetic analysis of block A did uncover some of the ancient relationships between *FT* genes, and highlighted the coexistence within a number of species and genera of paralogues originating from different ancestral lineages. These results are consistent with the pattern of ancestral duplications identified from whole genome analysis, which suggests that *Arabidopsis*, and probably all core *Brassicaceae* taxa, experienced three ancient whole-genome duplication (WGD) events leading to paleopolyploidy [50]. The most recent *At*-α (∼40 MYA) duplication was considered to explain the drastically increased species number in the core *Brassicaceae* [51]. The paleopolyploid *At*-α duplication was followed by later lineage-specific mesopolyploid WGD events. In Brassicaceae, at least four independent lineage-specific WGD events have been revealed and the whole genome triplication (*Br*-α) in *Brassica* was first proposed and then shown to have occurred before the radiation of the whole tribe *Brassiceae* [16], [18], [19], [52]. This triplication event further complicates analysis of the evolutionary relationships between different genera and species within the tribe *Brassiceae*, as shown by our results above.

### Transcript differentiation amongst *Brassica FT* genes

Duplicated genes may undergo three primary evolutionary fates, including silencing, sub-functionalization and neo-functionalization [53]–[55]. Within polyploids, and in particular allopolyploids, a wider repertoire may be available in terms of increased variation in dosage-regulated gene expression, altered regulatory interactions, and rapid genetic and epigenetic changes [56]–[58]. The ability to detect relative expression for all six *Brassica FT* copies is challenging due to high sequence similarity of transcripts, especially for the four *FT* paralogues within the duplication blocks of A7/C6 [35]. The lack of transcripts for *FT*-C2 in all accessions including *B. napus* and *B. oleracea* contrasted with the constitutive expression of *FT*-A2 in *B. rapa* and *B. napus* (Figure 3A–C). Moreover, whereas the four *FT* genes within the duplication block of A7/C6 were all silenced prior to vernalization in winter type *B. napus*, they were expressed in at least some spring type varieties (Figure 3A–B). These different expression patterns of *FT*-A2 might be attributed to the deletion or variation of FLC protein binding site within intron 1 of A2/C2 origin [10], [35], which would result in constitutive expression of *FT*-A2. However, silencing of *FT*-C2 may have resulted from TE insertion and consequent conserved high levels of DNA methylation in the TE and flanking sequences which are most found at CG and CHG sites. So, the TE itself and DNA methylation appears to have generated a functional pseudogene *FT*-C2, given the absence of expression in all materials analyzed. TEs are recognized as a primary target of cytosine methylation in eukaryotes, where it serves primarily to silence these repeat sequences [59]. DNA methylation is able to silence genes by blocking transcription initiation either by preventing protein binding or as a consequence of DNA methylation-induced chromatin remodeling [60]. In plants, TE methylation may occur adjacent to upstream regions where TEs have inserted, with ‘spreading’ of methylation then affecting *cis*-regulation, such as occurs in tomato, with the *CNR* gene affecting fruit ripening [61]. Although expression of *FT*-A7/C6 in *B. oleracea* appeared more abundant than in *B. rapa* (Figure 3C), and may compensate for the silencing of *FT*-C2, this requires further investigation. The difference in transcript abundance for *FT*-A7/C6 between winter and spring type *B. napus* under vernalization-free conditions may be caused by different control mechanisms for flowering within these two crop types, and in particular the interactions between FLC and FT. However, to clarify this, it is essential to sample leaf at multiple developmental time points from plants subject to vernalising and non-vernalising conditions.

Recent characterization of 12 *GPAT4* (sn-glycerol-3-phosphate acyltransferase 4) and seven *PSY* (Phytoene synthase) paralogues in *B. napus* and its two progenitors found that all were expressed, but with tissue specific expression exhibiting overlapping redundancy and signs of sub-functionalization [62], [63]. In contrast, the expression patterns of *FT* genes differed with developmental stage and in different species/cultivars, consistent with selection for different behaviors for this key regulator of the primary adaptive trait of flowering time, associated with the A and C genome divergence and more recent formation of *B. napus*. This behavior provides a wider range of options for adaption to different environments and domestication selection, such as the divergence between FT-A7/C6 which may correspond to the formation of winter and spring *B. napus* crop types. Interestingly, *FT*-A2 was constitutively expressed in all materials and developmental stages tested, and notably in winter-type *B. napus* before vernalization. *FT* genes have recently been found not only to regulate flowering but stomatal opening in *Arabidopsis*, vegetative growth in perennial poplar and storage organ formation in potato [64]–[66]. In sugar beet, two paralogues of *FT* have evolved antagonistic roles in the control of flowering, with the one functionally conserved with Arabidopsis *FT* being essential for flowering. However, the second paralogue represses flowering, with its down-regulation crucial for the vernalization response [67]. It is thus reasonable to suppose that the products of *FT*-A2 may participate in other physiological processes at least in winter-type *B. napus*, alternatively still in regulating flowering but in a different manner.

### Potential *FT* regulatory motif e in Brassicaceae

Three conserved blocks in the promoter region of *FT* have been found to be essential for activation by *CO* in *Arabidopsis*, with block A subsequently found as a *cis*-element for promoting gene expression, block B probably enhancing *FT* expression in response to blue light [68] and block C as a potential enhancer for long-distance genomic regulation in conjunction with *CO* [11]. Interestingly, we did not detect block B in the *Brassica FT*-A7/C6 lineage although these *FT* genes could be expressed normally (Figure 3A–B), indicating that block B is not required for *Brassica FT* activation. This may be explained by the shade avoidance regulation in *Arabidopsis* where the blue light is an important control of the shade avoidance syndrome responses [69]. However, for *Brassicas* this may not be an issue, given the ecological niches it occupies compared with *Arabidopsis*. We found two potential motifs for CO protein binding in *Brassica* block A (Figure 6A), each associated with the distinct *FT*-A2/C2 and *FT*-A7/C6 lineages, which may help to distinguish multiple *CO* paralogues in the *Brassica* genomes. Recently, another transcription factor, *PHYTOCHROME INTERACTING FACTOR4* (*PIF4*), has been identified that binds directly to the *FT* promoter near the transcript start site to regulate *FT* expression in *Arabidopsis* [70]. Another level of regulation of *FT* by *PIF4* may also exist in *Brassica* species which requires further investigation. We found that block C was significantly more conserved across the *Brassica* species and other species of Brassicaceae, even when located about 4.5–7.5 kb upstream of the *FT* start codon (Figure 5), suggesting an important role in *FT* regulation. This finding suggests it will be important to determine whether the potential motifs within block C are combined with other regulatory proteins or small RNA, which then interact with CO to promote *FT* expression.

## Materials and Methods

### Plant materials and growth conditions

Ninety-nine plant accessions representing diploid and tetraploid *Brassica* and a further nine non-domesticated Brassicaceae species were sampled, with DNA isolated from the leaves of plants grown in field or glasshouse (Table S1). No specific permits were required for the described field studies. Plants grown in the field at Huazhong Agricultural University were used solely for DNA extraction. *B. napus* plants of homozygous TapidorDH and Ningyou7DH were grown in soil in controlled environment (Sanyo Gallenkamp) at 18°C under LDs (16 h light/8 h dark) for 3 weeks, vernalized (8 h light/16 h dark) at 5°C for 7 weeks and 3 weeks, respectively, with half of the plants then transferred to LDs (16 h light/8 h dark, 18°C), and another plants to SDs (10 h light/14 h dark, 18°C) conditions.

The plants of *B. rapa* var. *trilocularis* R-o-18 and var. *pekinensis* Chiifu-401, and *B. oleracea* var. *alboglabra* Bailey were grown in cabinets (NK System Biotron, Tokyo, Japan) at 22° under LDs (16 h light/8 h dark). Tissue was collected from leaves at different growth stages (leaf 2, 6, 9 and 14) for transcript and DNA methylation analysis. Leaves were excised from the plant, the midrib removed, and the left hand side lamina used for RNA, the right hand side for DNA.

### Sequence acquisition and analysis

GenScan software optimized for Arabidopsis was used to identify protein-coding genes within TapidorDH BAC sequences [71]. *B. rapa* gene models were obtained from the Brassica database (BRAD) (http://brassicadb.org/brad/index.php) and coding sequences of *B. oleracea* and *B. napus* were compared to *B. rapa* and *Arabidopsis* genomes using Blastn and WU-BLAST against the BRAD and TIGR database (www.tigr.org/tdb/e2k1/bog1/). Transposable elements were predicted and located using “RepeatMasker” (http://www.repeatmasker.org). Primers used for isolating the *FT* upstream blocks and estimating the insertion events are given in Table S2. For TE insertion detection, the primer sets specific to the A2 lineage are successful when there is no TE insertion in block A and block B, whereas the primers specific to the C2 lineage amplify products when TE insertions are present. ClustalW was used for multiple alignments of the block sequences (http://www.ebi.ac.uk/). Phylogenetic trees were constructed using MEGA version 4.1 [72]. Bootstraps with 500 replicates were performed to assess node support.

### RNA isolation, reverse transcription and RT-PCR assays

Total RNA was extracted from leaves using the RNeasy Plant Mini Kit (Qiagen). cDNA was reverse-transcribed from total RNA (1 µg) with RevertAid™ First Strand cDNA Synthesis Kit (Fermentas) in a 20 µl reaction. Gene-specific primers were used for RT-PCR to detect Actin (30 cycles) and *Brassica FT* transcripts (30 cycles). The thermal cycling program was 94°C for 3 min followed by 30 cycles of 94°C for 30 s, annealing for 30 s, and extension at 72°C for 30 s, ending with a 10 min extension at 72°C. The primer sets used for RT-PCR detection are listed in Table S2.

### Bisulphite sequencing

DNA was extracted from leaves using the DNeasy Plant Mini Kit (Qiagen). 450 ng genomic DNA was subjected to two successive treatments of sodium bisulphite conversion using the EpiTect Bisulphite kit (Qiagen) according to the manufacturer's instructions. The reaction was then purified once more using the PCR purification kit (Qiagen). Forward (F) and reverse (R) primers for bisulphite sequencing PCR were designed using Kismeth (http://katahdin.mssm.edu/kismeth) and listed in Table S2. The control assays of ATP1 [73] were used to ensure the complete bisulphite treatment. PCR products were cloned into the pMD18-T vector (TaKaRa), and 10 individual clones were sequenced. Percentage methylation (% C) was calculated as 100×C/(C+T).

## Supporting Information

Table S1 **Plant materials used in this study.**
(XLS)

Table S2 **Description of primer sets used in this study and the resultant PCR products.**
(XLS)
